# Supplementary material for: ITRUSST consensus on standardised reporting for transcranial ultrasound stimulation
Source: Brain Stimul. Author manuscript; Available in PMC 2025 Sep 16. (PMC12436198; doi:10.1016/j.brs.2024.04.013)
Supplement: 1 [file NIHMS2003995-supplement-1.pdf]

Supplementary Material

A Linearity Assumption

We make the assumption that the ultrasound fields that this guide applies to are linear. Our rationale for assuming so is that the pressure values currently under investigation for the vast majority of transcranial ultrasound stimulation studies are low enough to be in the linear regime.

We note that linearity means that all or almost all of the energy is at the operating frequency, with minimal energy contained in the harmonics. Energy transferred to harmonic frequencies during nonlinear propagation of ultrasound results in an asymmetric pressure waveform, where the peak positive pressure becomes greater than the peak negative pressure. To verify linearity, the difference between the two pressure values can be examined. This can (and should) be checked by examining the frequency spectrum of the measured pressure waveforms and ensuring that e.g. the amplitude of the second harmonic is $\lesssim$5% of the amplitude of the fundamental frequency, or the difference between the peak positive and peak negative pressures is not more than $\sim$10%.

In our measurements, for a focused ultrasound transducer with a 64 mm radius of curvature and 64 mm aperture diameter operating in water at 500 kHz, the peak positive and negative pressure differ by >10% (indicating that the field is no longer quasi-linear) only when the pressure amplitude is >2 MPa. This corresponds to a Mechanical Index (MI) of >2.5. For a transducer with the same dimensions operating at 250 kHz, the peak positive and peak negative pressure differ by >10% only when the pressure amplitude is >3 MPa, for which the MI is 6.

When the field is linear, the size and position of the focal region for a given focal setting can be considered to remain constant between output levels. In addition, this assumption simplifies the calculation of several derived parameters in Section 5. For avoidance of doubt, throughout the guide, the term *pressure amplitude* is used to describe the amplitude of a sinusoidally varying pressure signal in steady-state under linear conditions, where the pressure amplitude is equal to the peak positive and negative pressures, and half the peak-to-peak pressure (see Fig. 2, Fig. B.1 for further details).

B Measurement of Free Field Acoustic Pressure Parameters

B.1 Guidance on Performing Measurements of Acoustic Pressure

Measurements of acoustic pressure fields are often made using a hydrophone scanned through the field using an automated scanning tank filled with deionised and degassed water. Measurement procedures and the choice of suitable sensors (usually hydrophones) and scanning equipment is specified in detail in international standards for the measurement of medical ultrasonic fields [1], with further specific definitions and measurement methods specifically for focusing transducers specified in [2]. Other sources of guidance and examples of good practice for hydrophone measurements of acoustic pressure fields can be found in the literature [3, 4, 5], and may be more accessible to readers of this document. Important factors to consider in order to reduce uncertainty in measurement include choice of hydrophone, alignment procedures, spatial sampling, and signal acquisition and processing.

B.2 Choice of Hydrophone

Measurements require a suitable sensor which has adequate sensitivity while minimising the effects of spatial averaging, is stable over time, and for the purposes of acquiring absolute pressure measurements, has a calibrated sensitivity (frequency dependent or at least at the frequency of interest). In general, the diameter or width of the hydrophone sensitive element should be less than one quarter of the wavelength of the acoustic field at the frequency of interest. In practice, at the low frequencies (< 1 MHz) often employed in TUS, the effective size of the hydrophone sensitive area can be significantly larger than the nominal size, increasing the effects of spatial averaging. For example, at 500 kHz, the wavelength of sound in water is $\sim$3 mm, so a hydrophone element diameter of less than 0.75

mm should be used. A hydrophone with a nominal element diameter of 0.5 mm may be suitable, although it should be noted that the effective element size may be significantly larger so it is strongly advised to choose a smaller element size [4, 6]. If spatial averaging is unavoidable and likely to be significant, corrections may be applied to the pressure measurements to compensate [7].

Most commercially available hydrophones can be expected to maintain stable sensitivity provided they are handled carefully and well maintained (i.e. the sensitive element should not be touched, they should not be exposed to pressures above the range of safe operation, they should be rinsed with DI water after use in tap water or other liquids to avoid build up of deposits on the element surface), and in most cases a soaking period of at least an hour should be allowed before measurements are made. Calibrated sensitivity at the frequency of interest traceable to international pressure standards can usually be provided by the hydrophone manufacturer, or obtained directly from a national measurement institute. Calibration at frequencies below 1 MHz may have to be specifically requested.

B.3 Uncertainty on Hydrophone Measurements

All hydrophone calibrations have an uncertainty which varies with frequency and calibration method. Hydrophone calibrations obtained from a national measurement institute (NMI) such as the National Physical Laboratory in the UK have a typical uncertainty of 9% in the range 250 kHz to 1 MHz. The uncertainty on hydrophone calibrations obtained from hydrophone manufacturers is typically approximately double that associated with NMI calibrations, up to 20% or more.

Other sources of systematic uncertainty include spatial averaging, which can become significant for large hydrophones in focused fields. Random uncertainty also arises from various sources including positioning, alignment and electrical fluctuations in amplifiers and source impedance. Despite the relatively high uncertainties, it has been demonstrated that reproducibility and repeatability of pressure measurements can be within 10% [8, 4], and is discussed further by Harris *et al.*[3]. It should also be noted that the uncertainty on pressure measurements will propagate to derived quantities. As intensity is proportional to the square of pressure, this results in a doubling of the uncertainty from pressure to intensity.

When comparing multiple pressure measurements of the same field, for example where sequential measurements are performed at intervals, or measurements are obtained from different sources, agreement is demonstrated if two measurements differ by less than the uncertainty on the measurements. Where multiple measurements agree to within the uncertainty, then an average value can be reported. If the difference is larger, it may indicate some change in performance of the device or error in the measurement process.

B.4 Signal Parameters and Acquisition

To achieve free field conditions under which the effects of reflections and interference are suppressed during measurements, the pulse parameters used for measurement of free field pressure parameters are usually different from those used during the study itself. The pulse length and timing of the signal waveform acquisition are chosen so that the measured field represents the field that would exist if the transducer was radiating into water continuously or with the comparatively long pulses used in TUS studies [9] . These timing conditions together with a water bath of sufficient volume or with a suitable absorbing lining ensures that interference from acoustic reflections is reduced or eliminated.

These quasi steady state conditions are achieved in a time window which starts after sound emitted from all parts of the transducer has reached all measurement locations, with additional time to allow for the ring-up time of the transducer, and ends before reflections from the measurement equipment reach the hydrophone (e.g. sound which is reflected from the hydrophone back to the source and then back to the hydrophone). The pulse duration should be chosen to be long enough to include some or all of this time window and to ensure that there is a section of the waveform which has a consistent amplitude. The pulse duration should be short enough to avoid overlap of electrical pick up from transducer drive voltage with the acquisition window, and reflections from other parts of the measurement set up. Most transducers have a ring up time of at least a few cycles at the beginning of the pulse. This

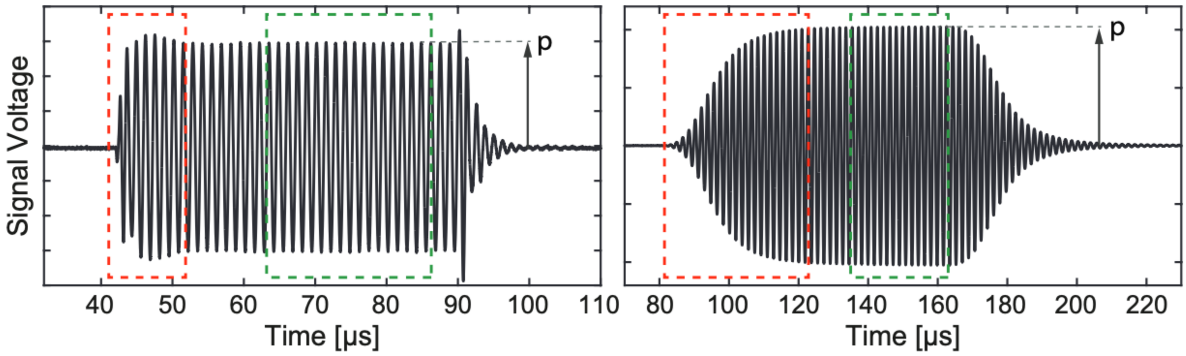

Figure B.1: Example focal waveforms measured at the position of spatial-peak pressure under free field conditions. The region within the red dashed boxes are the transducer ring up time before the waveform reaches a steady amplitude. The region within the green dotted boxes show the steady part of the waveform which should be used to calculate the pressure amplitude, denoted as p. The figure on the left shows a measurement from a transducer with a broad bandwidth (short ring-up time), and on the right a transducer with a narrow bandwidth (long ring-up time).

is the length of time it takes for the pulse amplitude to reach a steady value. Depending on the bandwidth of the transducer, this varies from a few cycles to 10s of cycles. Measurements of pressure amplitude should be made using part of the waveform which occurs after this time (see Fig. B.1).

Voltage signals from the hydrophone are usually acquired using an oscilloscope. The temporal sample rate should be sufficient to accurately represent the waveforms, with a recommended sample rate of at least 20 times the ultrasound frequency of interest [10]. The oscilloscope should be triggered using the trigger output from the drive system. This should provide a stable acquisition and provide the delay time between emission of the pulse from the transducer and arrival of the acoustic signal at the hydrophone. This can be used to calculate the axial position of the hydrophone relative to the transducer, given knowledge of the sound speed of water at the measurement temperature. It is recommended that this axial position calculation is performed at the focus of the field where the arrival time of the pulse can be most easily determined.

B.5 Alignment and Spatial Sampling

It is recommended that hydrophone measurements of the spatial distribution of acoustic pressure are performed in deionised and degassed water of known temperature, using an automated scanning tank with which the hydrophone can be positioned reproducibly to within 0.05 mm along three orthogonal directions. Following a rough alignment by eye, the hydrophone should be aligned to the beam axis by alternately scanning along the two lateral directions through the centre of the -6 dB focal region until the position of the centre is consistent to within approximately 100 μm. Finding the midpoint using the centre of mass of the -6 dB region rather than the peak value is recommended, since it is less susceptible to noise and fluctuations.

The transducer may be mounted in a fixed geometry using a mount that ensures that the beam axis is aligned with the parallel tank scanning axis, this should be verified. It is recommended that before acquiring pressure profile measurements, the hydrophone should be aligned to the spatial-peak pressure, to ensure that all line measurements pass through that through the focus. Where the transducer mount geometry is not fixed and adjustment of the angular position is possible, the transducer beam axis should be aligned with the parallel scanning axis. This can be achieved by aligning the hydrophone with the beam axis at two axial distances to determine any angular offset, and correcting the angular tilt and rotation of the transducer accordingly. Alternatively, where scans can be performed along arbitrary directions, the scan coordinates should follow the beam axis.

Measurements of the field should be made with adequate spatial sampling, of less than half a

wavelength or finer in order to accurately capture spatial features and peak pressure values. Rather than acquiring point measurements, it is recommended that orthogonal line scans are acquired along lines which pass through the location of the spatial-peak pressure. Once the hydrophone is aligned with the transducer beam axis, an axial line scan should be performed to identify the axial position of the spatial-peak pressure.

The axial position relative to the transducer can be estimated using the time of flight of the acoustic signal. However, there may be some error in this distance where there are e.g. time delays between the trigger signal and generation of the acoustic output due to the system electronics. Additionally, where the transducer construction includes lenses or coupling media, the measurement coordinates are often defined relative to a reference plane which is not the origin of the acoustic signal. In this case, a physical measurement of the reference plane to hydrophone distance should be made. This can be done using e.g. a force sensor, dummy hydrophone, or offset block.

B.6 Signal Processing

Hydrophones output a voltage signal which must be converted to pressure using the known hydrophone sensitivity. Some signal processing steps should be taken to ensure that errors are not introduced during this conversion. For linear fields, the peak negative pressure, peak positive pressure and the pressure amplitude can be expected to be equivalent. Peak negative pressure is usually given as a quantity linked to mechanical effects. The values can be obtained by several methods, but it is recommended that a method is chosen which reduces the impact of noise and fluctuation of the signal amplitude throughout the acquisition window.

For quasi steady state signals, the pressure amplitude can be acquired by performing a fast Fourier transform (FFT) on a measured waveform which has been trimmed to include a whole number of cycles from a steady part of the waveform, then extracting the magnitude of the FFT at the frequency of interest. The length (number of samples) in the voltage signal should be large enough to provide adequate sampling in frequency space so that the amplitude at the frequency of interest can be obtained. Alternatively, the minimum value of each cycle from a steady part of the acquired waveform can be obtained and averaged. The peak negative voltage or voltage amplitude can then be converted to pressure by dividing by the hydrophone sensitivity at the frequency of interest given in units of V/Pa, or by deconvolution of the hydrophone response from the voltage waveform [1, 3, 8].

B.7 Scaling Spatial-Peak Pressure Measurements by Drive Level

The free field spatial-peak pressure amplitude at each of the study output level settings should be reported. The pressure can be obtained either directly from a measurement with the transducer operated at the study output level and focal setting, but in practice, it may be simpler and more practical to establish a scaling from the measurement output level, or the output level given in the manufacturer's test report, to the study output level(s) for a given focal setting.

While the acoustic pressure field is linear, the output pressure of most systems is expected to scale linearly with the voltage applied to the transducer. So for example, focal pressure amplitude should scale linearly with drive voltage amplitude. Correspondingly, if the output setting used is power or intensity, since this is proportional to pressure squared, the spatial-peak pressure should scale linearly with the square root of the power or intensity setting. It is assumed that at the range of output levels used for TUS, the location and volume of the focal region will remain constant provided no changes are made to the focal settings.

The relationship between output level setting and spatial-peak pressure should be verified if possible by making several measurements of the spatial-peak pressure at different output level settings for a given focal setting. A line fitted to the measurement points can then be used to obtain the pressure for any output level setting later used in studies. Note that if the focal position setting is changed, a different scaling is needed.

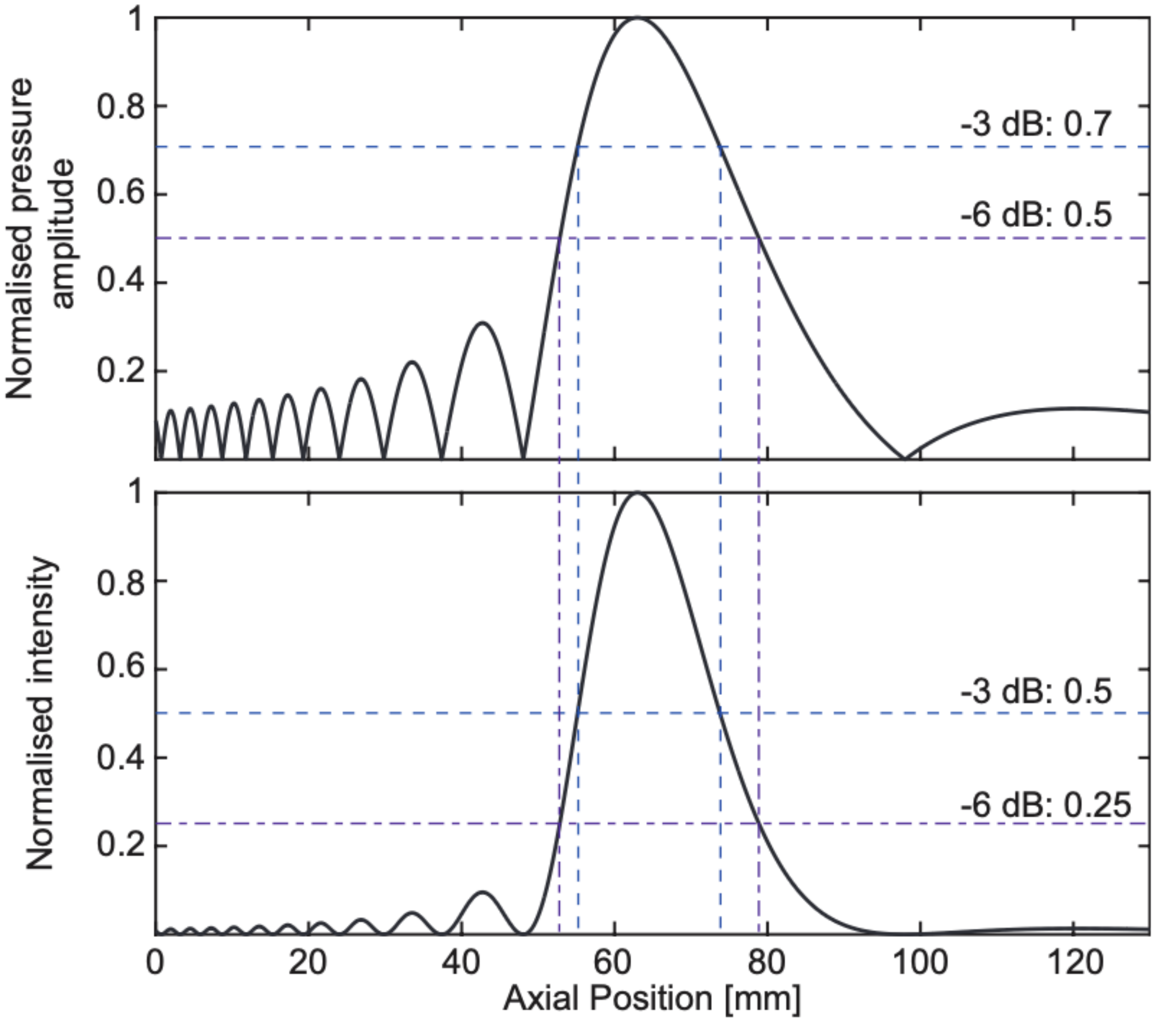

Figure B.2: Axial profiles of normalised pressure amplitude calculated using O'Neil's solution [11] (top) and corresponding normalised intensity (bottom), showing the -3 dB and -6 dB axial focal widths which correspond to different fractional thresholds in pressure and intensity.

B.8 Decibels and Focal Size Quantities

In acoustics, decibels are used to describe the loudness of sound on a logarithmic scale, as the range of pressure amplitudes in the human audible range is large, similarly for the range of pressure amplitudes in the transmitted and received pulses in ultrasound imaging. When used in ultrasound field characterisation, they are often used to describe a change in or ratio of pressure or intensity. These ratios in units of decibels [dB] are calculated from the log to the base 10 of the ratio of the pressure or intensity at the point of interest, p, to the reference pressure or intensity, p_{ref}:

$$10\,\log_{10}\left(\frac{I}{I_{ref}}\right)=10\,\log_{10}\left(\frac{p^2}{p_{ref}^2}\right)=20\,\log_{10}\left(\frac{p}{p_{ref}}\right)\;[dB] \qquad (1)$$

In the context of determining focal width values, the reference pressure or intensity is the spatial-peak value. The same focal width value can be calculated from either the pressure or intensity profiles in terms of dB, with the appropriate fractional threshold (see Fig. B.2). The full width half maximum of the intensity profile is the -3 dB region, but in terms of pressure, this -3 dB region corresponds to the region over which the pressure exceeds 70% of the maximum value. Note that these quantities are useful for comparison of fields in water or for looking at focal distortion in situ, for example, but

since they are relative quantities, they do not indicate the region of potential effect, since this is likely to correspond to an absolute pressure threshold.

B.9 Obtaining Pressure Quantities from Manufacturer's Report

It is expected that the manufacturer should provide some conversion between the setting used to control the output level and the spatial-peak pressure for at least a subset of focal settings. It may not be possible to obtain all required parameters for all possible focal settings from the manufacturers report, but all parameters that can be obtained should be reported. For example, if the position setting refers to the centre of the focal region rather than the location of the spatial-peak pressure, then it may not be possible to report both for every focal setting used. The difference between these positions will vary depending on the transducer parameters and focal settings, it may be as much as 10 or 20 mm.

C Reporting Average Parameters

In some cases where a study includes a large number of participants, each with personalised ultrasound parameters, it could be impractical to report the free field parameters, drive system settings, and estimated *in situ* exposure parameters under each condition used. In this situation, it is appropriate to report the mean, minimum and maximum values.

In the simplest case, the same drive system settings and free field pressure parameters may be applied for all participants, but individualised *in situ* exposure parameters obtained from simulations using the participants anatomy. In this case, it is reasonable to report the mean, minimum and maximum of the estimated *in situ* exposure parameters across all participants.

In another the case, the drive system settings may be adjusted with aim of delivering the same ultrasound pressure to the same brain region across subjects. In this case, the range of drive system settings used should be reported. The mean, minimum and maximum of the free field spatial-peak pressure amplitude and lateral and axial width of the focal volume should be reported, and the range of the positions of the free field spatial-peak pressure amplitude.

Where an array transducer is used with steering and aberration correction to obtain the same *in situ* spatial-peak pressure amplitude across subjects, free field measurements made under the exact conditions are not relevant and would be impractical to perform for every condition. In this case, measurements of the free field pressure parameters should be made at the geometric focus or central position, and over the steering range employed during the study. Free field spatial-peak pressure amplitudes lateral and axial width of the focal volume measured across the steering range relative to the geometric focus or central position will illustrate the steering and focusing performance of the array. The range of drive system settings used should also be reported. The range of *in situ* lateral and axial widths of the focal volume obtained from simulations used to perform the aberration correction should be reported, since these may vary depending on position and skull anatomy.

D Additional Experimental Reporting

There are many other aspects to the TUS experiment that critically impact the experiment and should be reported. Due to the large number of different measurement conditions, equipment, and experimental setups, it is not possible to be prescriptive about how these should be reported. Here, we briefly list other aspects of the TUS experiment that fall outside of the scope of this paper but are also important in fully describing a study.

- Hair preparation
- Subject positioning (e.g. sitting or lying) and fixation (e.g. chin rest)
- Transducer alignment and fixation
- Neuronavigation equipment and procedure

- Auditory perception
- Masking and earplugs
- Sham conditions and blinding
- Environmental conditions (e.g. background noise and light levels)
- Additional measurement equipment
- Subject experience, side effects, and adverse events
- Any safety and electromagnetic compatibility tests performed

References

[1] "IEC 62127-1:2022: Ultrasonics — Hydrophones - Part 1: Measurement and characterization of medical ultrasonic fields up to 40 MHz," 2022. International Electrotechnical Commission, Geneva, Switzerland.

[2] "IEC 61828:2001: Ultrasonics — Focusing transducers — Definitions and measurement methods for the transmitted fields," 2001. International Electrotechnical Commission, Geneva, Switzerland.

[3] G. R. Harris, S. Howard, A. Hurrell, P. A. Lewin, M. E. Schafer, K. A. Wear, V. Wilkens, and B. Zeqiri, "Hydrophone measurements for biomedical ultrasound applications: A review," *IEEE Trans. Ultras., Ferroelectr. Freq. Contr.*, pp. 1–1, 2022.

[4] E. Martin and B. E. Treeby, "Investigation of the repeatability and reproducibility of hydrophone measurements of medical ultrasound fields," *J. Acoust. Soc. Am.*, vol. 145, no. 3, pp. 1270–1282, 2019.

[5] Preston, ed., *Output measurements for medical ultrasound*. Springer-Verlag, 1991.

[6] K. A. Wear and A. Shah, "Nominal vs. actual spatial resolution: Comparison of directivity and frequency-dependent effective sensitive element size for membrane, needle, capsule, and fiber-optic hydrophones," *IEEE Transactions on Ultrasonics, Ferroelectrics, and Frequency Control*, pp. 1–1, 2022.

[7] K. A. Wear, "Spatiotemporal deconvolution of hydrophone response for linear and nonlinear beams—part i: Theory, spatial-averaging correction formulas, and criteria for sensitive element size," *IEEE Trans Ultrason., Ferroelectr., and Freq. Control*, vol. 69, pp. 1243—1256, 2022.

[8] Y. Liu, K. A. Wear, and G. R. Harris, "Variation of high-intensity therapeutic ultrasound (HITU) pressure field characterization: Effects of hydrophone choice, nonlinearity, spatial averaging and complex deconvolution," *Ultrasound in Medicine and Biology*, vol. 43, no. 10, pp. 2329–2342, 2017.

[9] "IEC TS 62556: Ultrasonics - Field characterization - Specification and measurement of field parameters for high intensity therapeutic ultrasound (HITU) transducers and systems," 2014. International Electrotechnical Commission, Geneva, Switzerland.

[10] "IEC 62127-2:2007+A1:2013: Ultrasonics — Hydrophones - Part 2: : Calibration for ultrasonic fields up to 40 MHz," 2013. International Electrotechnical Commission, Geneva, Switzerland.

[11] H. O'Neil, "Theory of focusing radiators," *The Journal of the Acoustical Society of America*, vol. 21, no. 5, pp. 516–526, 1949.
